# Effects of water level regulation on the seed germination and production of annual plant *Xanthium sibiricum* in the water-level-fluctuating-zone of Three Gorges Reservoir

**DOI:** 10.1038/s41598-017-04599-4

**Published:** 2017-07-11

**Authors:** Jianhui Liu, Feng Lin, Shaohua Shi, Qiaoli Ayi, Songping Liu, Bo Zeng

**Affiliations:** Key Laboratory of Eco-environments in Three Gorges Reservoir Region (Ministry of Education), Chongqing Key Laboratory of Plant Ecology and Resources Research in Three Gorges Reservoir Region, School of Life Sciences, Southwest University, Chongqing, 400715 China

## Abstract

Vegetation effectively slows down the deterioration rate of the ecosystem in the water-level fluctuation zone (WLFZ). In this study, we investigated the impact of impoundment on the germination of seeds from *Xanthium sibiricum* fruits with various degrees of maturity and produced by *X*. *sibiricum* plants growing at different elevations in the WLFZ. Seed production by *X*. *sibiricum* under the current water level regulation mode was also investigated. Seeds were collected from seven elevations of the WLFZ of Three Gorges Reservoir (TGR) and exposed at these elevations to submergence. Seed production of the plants germinated from *X*. *sibiricum* seeds was observed. The maturity degree of *X*. *sibiricum* fruits from different elevations has no influence on the seed production by the plants that are produced from the fruits. Under the present water-level regulation mode, *X*. *sibiricum* growing above 155 m are able to generate mature seeds and thus provide seed sources for population maintenance, although the plants growing at an elevation below 165 m failed to produce fruits in 2010 due to unusual flooding. This study is useful for the vegetation recovery and reconstruction in other regions with hydrological characteristics similar to that of the TGR.

## Introduction

The regulation of river flow through the construction of dams for hydroelectric power generation, irrigation and flood control has been a common phenomenon worldwide for centuries but has shown increasing intensity in the last century^1^. Dams create a series of eco-environmental problems while providing benefits to humanity; in particular, they cause damage to the structure and functions of water level fluctuation zones (WLFZs)^2–4^. Because the artificial regulation of reservoir impoundment has disrupted the original water level variation rhythm of rivers, large areas of alternating wet-and-dry drawdown zones with water-level fluctuations are formed along river banks^5,6^. The original terrestrial vegetation in the WLFZ, which changes unavoidably due to changes in hydrologic conditions, has been impacted or even destroyed^7,8^. Therefore, the ecological restoration of the vegetation in the WLFZ has attracted the attention of scholars.

The Three Gorges Dam in China is one of the largest dams in the world, with a 350-km^2^ WLFZ in the Three Gorges Reservoir (TGR). Since the construction of the dam, the dynamics of the vegetation in the WLFZ has been constantly traced, but the ecological restoration in the WLFZ remains in the exploration stage^9–13^. Under the water level regulation mode “impounding in winter and draining in summer”, the vegetation in the WLFZ of the TGR degenerates seriously due to long-duration, deep, and anti-season submergence^10^. The TGR starts to impound water every year in September, and the water level of the TGR then increases gradually to the maximum level of 175 m, at which it is maintained for several months. Starting at December every year, the water level gradually falls to the minimum level of 145 m. During the flood season, which occurs between June and September, the water level remains at a constant 145 m. Therefore, the periodic rise and fall of the water level produce a drawdown zone with a vertical height difference of 30 m^11^. Since the impoundment in 2003, the vegetation growing in the WLFZ has changed substantially, and the dominance of annual plants has significantly increased. A community composed of annual plants has gradually become dominant in the WLFZ^11–14^.

Relying on the vegetation and seed bank available in the drawdown zone to achieve natural restoration is a time-saving, efficient, and economically practical approach^15,16^. Whether an annual can grow and reproduce naturally in the WLFZ in the TGR primarily depends on the completion of two processes in its life history: (1) seed germination, i.e., whether seeds produced prior to impoundment can tolerate long-term and deep complete submergence in the WLFZ and germinate successfully after water recession, and (2) seed generation, i.e., whether the plants in the WLFZ can generate a sufficient number of mature seeds prior to impoundment, thus providing sufficient seeds for population persistence. Annual plants can exist naturally in the TGR WLFZ only when these species can successfully complete these two life processes.

Under the current water level scheduling mode, the impounding and receding processes last as long as several months, which leads to substantial differences in the degree of submergence (including submergence depth, starting and stopping season, and submergence duration) of the seeds of plants growing at different elevations and in the reproduction time of the plants initiated by seed germination. Compared with those growing at higher elevations, annual plants growing at a lower elevation are flooded earlier in the year and at deeper depths by impoundment every year and exposed to air later in the year by water withdrawal. The seeds of the plant population grown at a lower elevation are flooded longer by impoundment, and these plants have less time for growth and reproduction after water recession compared to the plants at higher elevations. A higher degree of submergence might cause seeds to be unable to germinate normally, whereas a shorter growth and reproduction time means that plants might not produce mature seeds, ultimately resulting in elimination of the annual plant population naturally growing at this elevation.

*Xanthium sibiricum*, an annual plant that was originally sparsely distributed in the WLFZ, has become one of the dominant plants in the zone, particularly in the WLFZs of some tributaries of the Yangtze River, and extensive areas of *X*. *sibiricum* vegetation have been formed^17–19^. *X*. *sibiricum* is a tall annual herbaceous plant with the ability to adapt to the environment. A vegetation belt with abundant *X*. *sibiricum* plays a central role in the current management of soil and water conservation and ecosystem protection in the TGR WLFZ, where vegetation destruction is serious. The natural and persistent *X*. *sibiricum* vegetation not only achieves a certain degree of natural vegetation recovery in the area but also effectively slows down the deterioration of the ecosystem in the area. In a field survey conducted in the TGR WLFZ, we found that plants in *X*. *sibiricum* communities growing at different elevations in the WLFZ show different degrees of maturity before the impoundment, and the maturity degree of the seeds produced by *X*. *sibiricum* plants also differs. Therefore, we asked the following questions: Can all of these seeds, which are of different degrees of maturity, tolerate submergence in the WLFZ? Will the plants formed from these seeds be able to produce mature seeds before submergence by the next impoundment? Additionally, will the maturity degree of these seeds of *X*. *sibiricum* affect the generation of seeds by the subsequent round of plants?

To explore these questions, this study investigated the germination of seeds of *X*. *sibiricum* and the plant’s seed production capacity at different elevations in the TGR WLFZ. We explored the potential applications of *X*. *sibiricum* for vegetation restoration and reconstruction in the TGR WLFZ. The answers to these questions will help us gain a deeper understanding of the growth of annual plants in the WLFZ of large-scale reservoirs and form a theoretical basis for the use of annual plants in vegetation recovery and reconstruction in the WLFZ of large-scale reservoirs.

## Results

### Impact of submergence on the germination of *X. sibiricum* seeds

#### Germination rates of unsubmerged seeds of different maturity states

**Figure 1:**
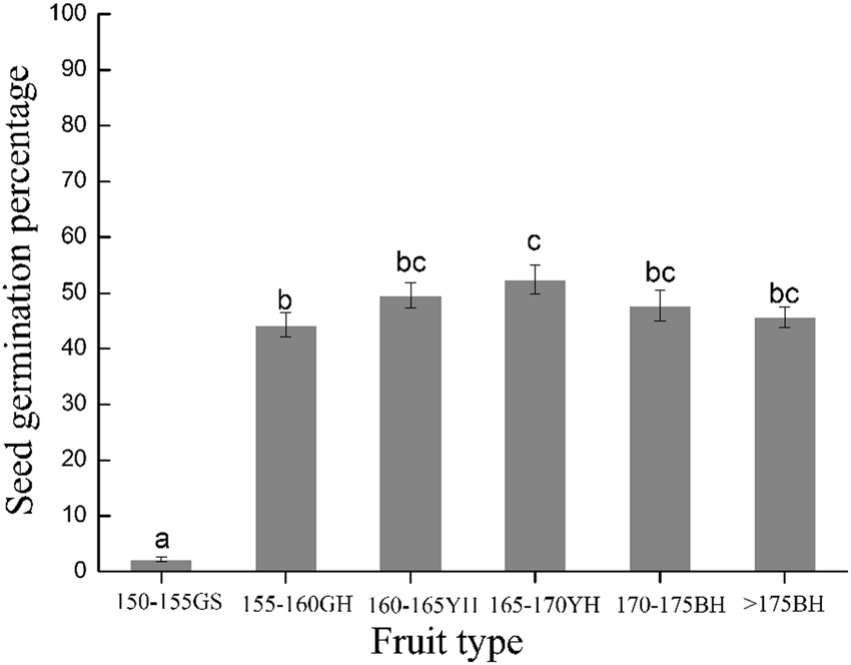
Germination rates (means ± SE) of *Xanthium sibiricum* seeds unsubmerged.

The results regarding the natural germination capacities of *X*. *sibiricum* seeds unsubmerged were the following: The seed germination rate of GS fruits was significantly lower than those of GH, YH, and BH fruits (*P* < 0.05), whereas no significant differences in the seed germination rate were found among GH, YH and BH fruits (Fig. 1). The maturest states of the fruits collected at elevations of 150–155 m, 155–160 m, 160–165 m, 165–170 m, 170–175 m, and >175 m were GS, GH, YH, YH, BH, and BH, respectively (Table 1), and their germination rates were 2.225%, 45.25%, 49.65%, 52.45%, 47.75%, and 45.625%, respectively. The germination rate of the GS fruits collected from the elevation of 150–155 m was significantly lower than that of the fruits collected at elevations higher than 155 m (*P* < 0.05), whereas no significant differences were observed among the fruits collected at elevations higher than 155 m (*P* > 0.05).

**Table 1:** Fruit collecting time and fruit maturity state at various elevations at the WLFZ of TGR.

Collection elevation (m)	Collection time	Fruit maturity state
145–150	2009.09.04	No fruits
150–155	2009.09.18	GS
155–160	2009.09.28	GH
160–165	2009.10.07	YH
165–170	2009.10.15	YH
170–175	2009.10.26	BH
>175	2009.10.26	BH

#### Impact of submergence on the seed germination rates of X. sibiricum fruits of different maturity ﻿states

**Figure 2:**
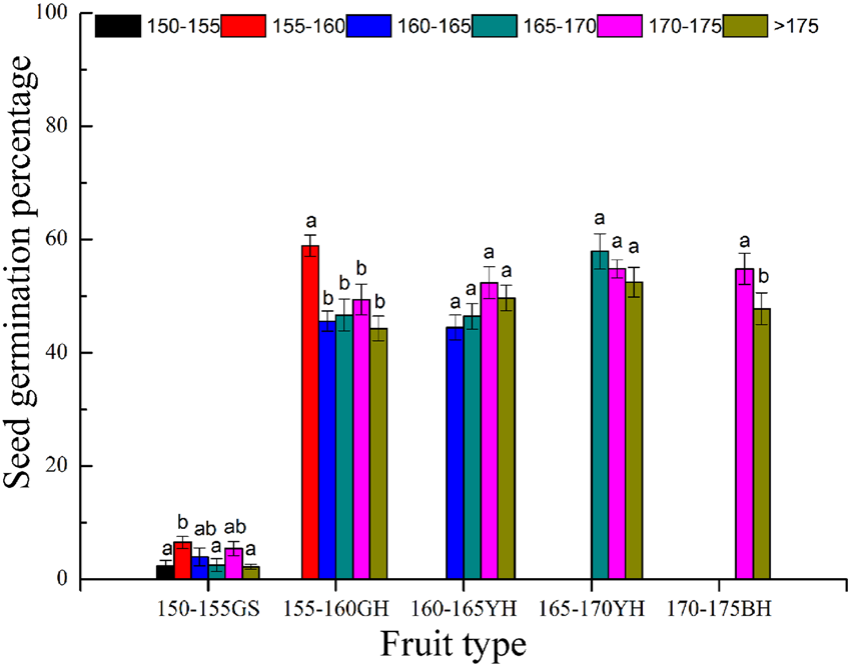
Seed germination rates (means ± SE) of fruits of different maturity states desubmerged at different elevations. Different lowercase letters indicate significant difference at the 0.05 level.

The germination rates of the seeds at different elevations undergoing submergence were not lower than that of the seeds unsubmerged (>175 m, without submergence) (Fig. 2). The germination rates of the GS seeds collected at an elevation of 150–155 m were all lower than 5%. With the exception of the 155–160 m elevation, at which the GS seeds showed a higher germination rates than those at >175 m (P < 0.05), other elevations did not show such a significant difference compared with the >175 m group (P > 0.05). The germination rates of seeds collected at 155–160 m were higher than 45% at different elevations. The seeds collected at different elevations did not show significant differences, with the exception that the seeds collected at 155–160 m showed a germination rate of 59%, which is higher than that of the fruits collected at other elevations (P < 0.01). The seeds collected from the 160–165 m and 165–170 m elevations did not show significant differences at different elevations. The BH seeds collected from the 170–175 m elevation showed a significantly higher germination rate than the control seeds (P < 0.05).

#### Impact of submergence on the germination course of X. sibiricum seeds from fruits of different maturity states

**Figure 3:**
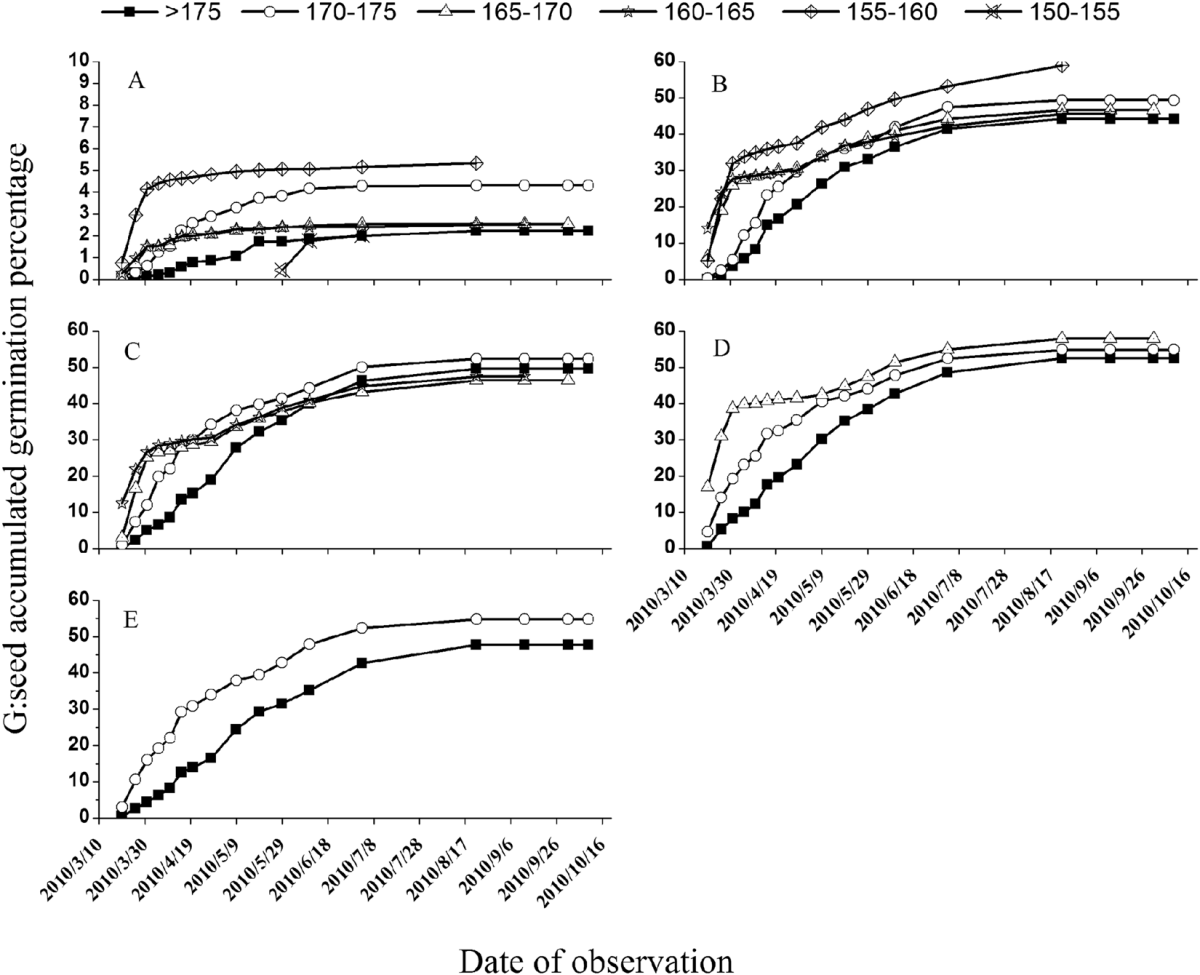
Germination course of seeds of different maturity states placed at different elevations. The symbols ‘◾’, ‘⚬’, ‘Δ’, ‘ж’, ‘⬦’ and ‘◽’ represent the elevations at which the seeds were submerged, >175 m, 170–175 m, 165–170 m, 160–165 m, 155–160 m, and 150–155 m, respectively. A, GS seed germination course at 150–175 m. B, GH seed germination course at 155–175 m. C, YH160–165 seed germination course at 155–175 m. D, YH165–170 seed germination course at 165–175 m. E, seed germination course of BH170–175 at 170–175 m.

The germination dynamic curves of the seeds collected from the same elevation after being submerged with different intensities were shifted upward compared with that of the unsubmerged seeds (Fig. 3). With the exception of the GS seeds from 150–155 m, whose germination did not show significant differences after submergence of different intensities, the seeds from the other elevations showed a significant difference in germinability during the first 45 days of the germination phase compared with their corresponding control (P < 0.05; Table 2). The germinability of the seeds produced from different elevations but subjected to submergence at the same elevationshowed the following trend: 150–155GS < 155–160GH, 160–165YH, 170–175BH, 175BH < 165–170YH.

**Table 2:** Germination of seeds collected from different elevations during the first 45 days of germination course after being submerged at different elevations in the water level fluctuation zone of the TGR (means ± SE).

Elevation (m)	Fruit types					
	150–155GS	155–160GH	160–165YH	165–170YH	170–175BH	> 175BH
145–150	—	—	—	—	—	—
150–155	2.03 ± 0.38a	—	—	—	—	—
155–160	4.94 ± 0.69bA	41.88 ± 1.42aB	—	—	—	—
160–165	2.17 ± 0.51 aA	36.9 ± 1.58bB	36.25 ± 1.33abB	—	—	—
165–170	2.3 ± 0.98 aA	36.5 ± 2.28bB	35.85 ± 1.63abB	44.8 ± 1.72aC	—	—
170–175	3.72 ± 0.83abA	36.08 ± 2.06bBC	39.8 ± 1.67bBC	42.05 ± 0.88aC	39.53 ± 1.34aB	—
>175	1.73 ± 0.34 aA	30.98 ± 1.79cB	32.25 ± 1.17aBC	35.2 ± 1.61bC	29.3 ± 1.45bB	30.63 ± 1.32B

Lowercase, seeds from the same source that were placed at different elevations. Uppercase, seeds from different sources that were placed at the same elevation. Identical letters, P > 0.05. Different letters, P < 0.05.

### Seed production of *X. sibiricum* plants at different elevations

**Figure 4:**
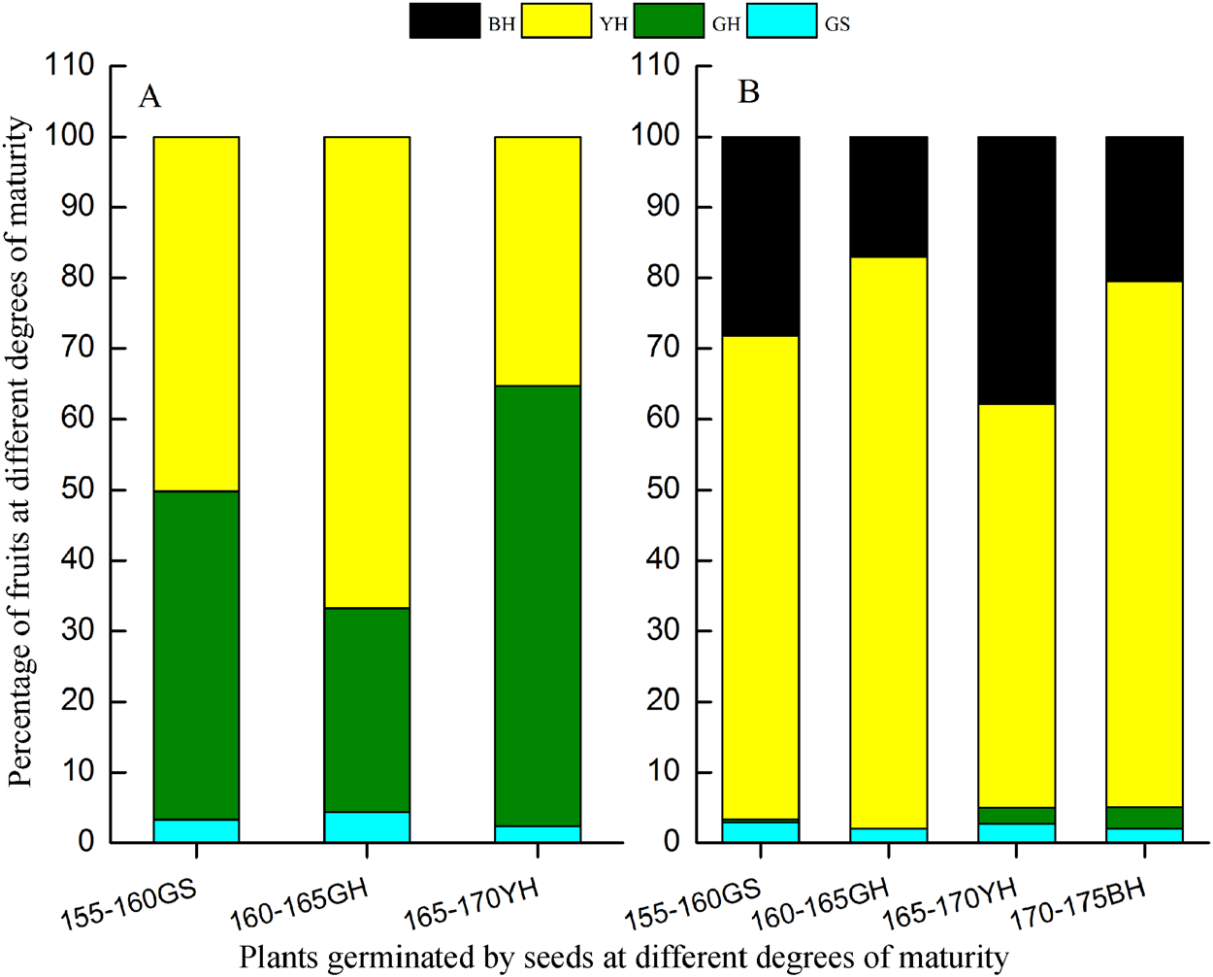
Percentage of fruits at different maturity states produced in plants growing at different elevations. A, Plants at 165–170 m. B, Plants at 170–175 m. Different colors indicate fruits of different maturity states.

The water level in the WLFZ during the summer in 2010 reached 165 m. For this reason, the *X*. *sibiricum* plants growing at 150–165 m had no fruit production by the impoundment of the reservoir in September, 2010. The *X*. *sibiricum* plants generated from GS, GH and YH fruits and grown at 165–170 m all produced more than 180 fruits per plant respectively, with no significant difference between plants of different orgins (P > 0.05; Table 3). For any plant growing at 165–170 m, the proportion of fruits with maturity states﻿ of GH and YH was higher than 90% (Fig. 4A). The plants generated from GS, GH, YH and BH fruits and grown at 170–175 m all produced more than 300 fruits per plant respectively﻿, and the fruit production of plants of GS origin was not different from those plants of GH and YH origins (P > 0.05) (Table 3). The proportion of fruits with maturity states of GH, YH and BH in any plant growing at 170–175 m were higher than 95% (Fig. 4B), and the fruit production of plants growing at 170–175 m was clearly greater than that of plants at 160–165 m (P < 0.01) (Table 3).

**Table 3:** Fruit yield of plants which were generated from seeds in fruits of different maturity states ﻿and grown ﻿﻿at different elevations.

Elevation (m)	Fruit types from which plants were generated	Fruit yield per plant
150–165	GS	0
	GH	0
165–170	GS	203 ± 31a
	GH	200 ± 25a
	YH	185 ± 30a
170–175	GS	309 ± 29b
	GH	337 ± 19bc
	YH	346 ± 38bc
	BH	382 ± 25c

Different lowercase letters indicate a significant difference at the 0.05 level.

## Discussion

The TGR is one of the largest reservoirs in the world, and the original vegetation in its WLFZ was extirpated by the reservoir’s unseasonal submergence, including extreme depths and long duration. Vegetation, as an integral component of the WLFZ, has important ecological functions, such as damming, receiving pollution, water and soil conservation, windbreaking and protection from wave action. Vegetation restoration requires a lengthy effort. Under the current water level scheduling mode, whether the extant annual *X*. *sibiricum* plant communities can enjoy a continuing presence in the zone mainly depends on the submergence-tolerance ability of fruits at different degrees of maturity and the production of seeds generated by *X*. *sibiricum* plants during the limited exposure period.

The maturity degree of a seed has a direct influence on its germination and anti-adversity ability^14^. The normal development of seeds involves the processes of husk development, embryo development, embryo dehydration, and husk dehydration, and only the seeds that complete the embryo development process have the capability to germinate, whereas dehydration of the embryo and husk play important roles in the anti-reversion force of the seeds. For example, the maturation process of legume seeds generally involves five stages, namely the green ripe stage, the early yellow ripe stage, the late yellow ripe stage, the full ripe stage and the dead ripe stage. In the green ripe and early yellow ripe stages, the seed volume has reached its maximum, the seed contains a high level of water, the husk is soft, and the seed cannot germinate normally. In contrast, in the late yellow ripe stage, the seed begins to undergo dehydration, the husk gradually hardens, and the seed volume shrinks significantly. At this stage, the seed becomes hard, has relatively high germination and anti-reversion capacities, and subsequently begins to germinate normally^20,21^. During development of the *X*. *sibiricum* achene, the husk develops first, and the fruit reaches the GS status. The seed then develops gradually to enrich the achene, and the fruit shows a GH status. Husk dehydration then occurs, and the husk turns from green to yellow. After completion of the dehydration step, the fruit shows a black hard appearance. In this study, the GS fruits were found to have a low germination capacity, which is presumably due to the incomplete internal development of the seeds at this degree of maturity, even though the fruit shows a rather complete appearance. In addition, our results on the germination rates of *X*. *sibiricum* fruits at different elevation without undergoing submergence showed that the germination abilities of the green and yellow hard seeds were comparable to that of the black hard seeds.

The BH fruits of *X*. *sibiricum* have a strong tolerance to flooding^22^. In this study, the germination rates of the seeds at the same elevation after submergence at different elevations were not lower than that of the control, regardless of their maturity (Fig. 2). Furthermore, submergence accelerated the germination process of the seeds (Fig. 3) and improved their germination energy at the early germination stage (Table 2). These results indicate that the seeds from different elevations and at different degrees of maturity all show strong tolerance to submergence. This phenomenon might be due to the outer husk of the *X*. *sibiricum* achene. The damage to the seed resulting from submergence is most likely the result of exudation of the embryo constituents, which include salt, protein and starch, under submergence. The resulting damage to the embryo influences the later development of the plants^23^. The *X*. *sibiricum* seed husk can make the inner seed impervious to damage under submergence stress and does not change the seed germination percentage. The thick and hard husk is only weakly permeable to air and water, and this structure prevents the exchange of water and air to some extent and inhibits seed germination^22,24^. A storage experiment conducted by Shen *et al*. showed that the *X*. *sibiricum* husk is a major factor limiting seed germination. The germination percentage of seeds whose husk was removed was clearly higher than that of those seeds with a husk. Additionally, storage in wet sand can soften the husk of the *X*. *sibiricum* seed, promote water permeability and improve the seed germination percentage^20^. After the *X*. *sibiricum* fruits at different degrees of maturity used in this study underwent submergence for different lengths and at different depths, the course of seed germination was clearly accelerated. It is likely that the soaking resulting from flooding softened the husk and seed coat of *X*. *sibiricum*, thereby increasing its permeability and enhancing seed germination. Submergence promoted more rapid and earlier germination of *X*. *sibiricum* seeds, enabling *X*. *sibiricum* plants to have more time for vegetative growth, flowering and reproduction and therefore improving the chances of the plants to produce seeds successfully before the impoundment. This outcome facilitates the long-term persistence of *X*. *sibiricum* populations in the TGR WLFZ.

The life cycle and rhythms of annual plants are relatively fixed: Under specific seasonal conditions, their seed phase, growth and production durations are relatively fixed^21^. Within the limited growth window at different elevations in the TGR WLFZ, *X*. *sibiricum* has to produce seeds with high tolerance to submergence and a strong germination ability before annual submergence in order to guarantee its long-term natural continuation. Combined with the results obtained in this study, *X*. *sibiricum* can ensure its natural continuation at different elevations only of it produces fruits with a maturity of at least the green hard level (Fig. 1).

Under the current water level scheduling model of the TGR, *X*. *sibiricum* growing at elevations lower than 155 m cannot produce mature seeds (Table 1) because an overly long submergence duration and a too short air exposure time impeded completion of the growth and production processes of the plant. In contrast, the plants growing at elevations above 155 m could complete their normal growth and production due to a sufficient exposure time (Table 1 and Fig. 1). Furthermore, the degree of maturity of *X*. *sibiricum* fruits did not have an obvious influence on the seeds that generated later. The fruit yield of *X*. *sibiricum* plants germinated from seeds of *X*. *sibiricum* fruits at different degrees of maturity placed at 165–170 m and 170–175 m and the ratios of fruits at different degrees of maturity to the total fruits showed no difference (Table 3 and Fig. 4). The fruit yield of plants at 170–175 m and the ratio of mature fruits to total fruits were significantly higher than those of plants at 165–170 m due to a longer exposure time.

**Figure 5:**
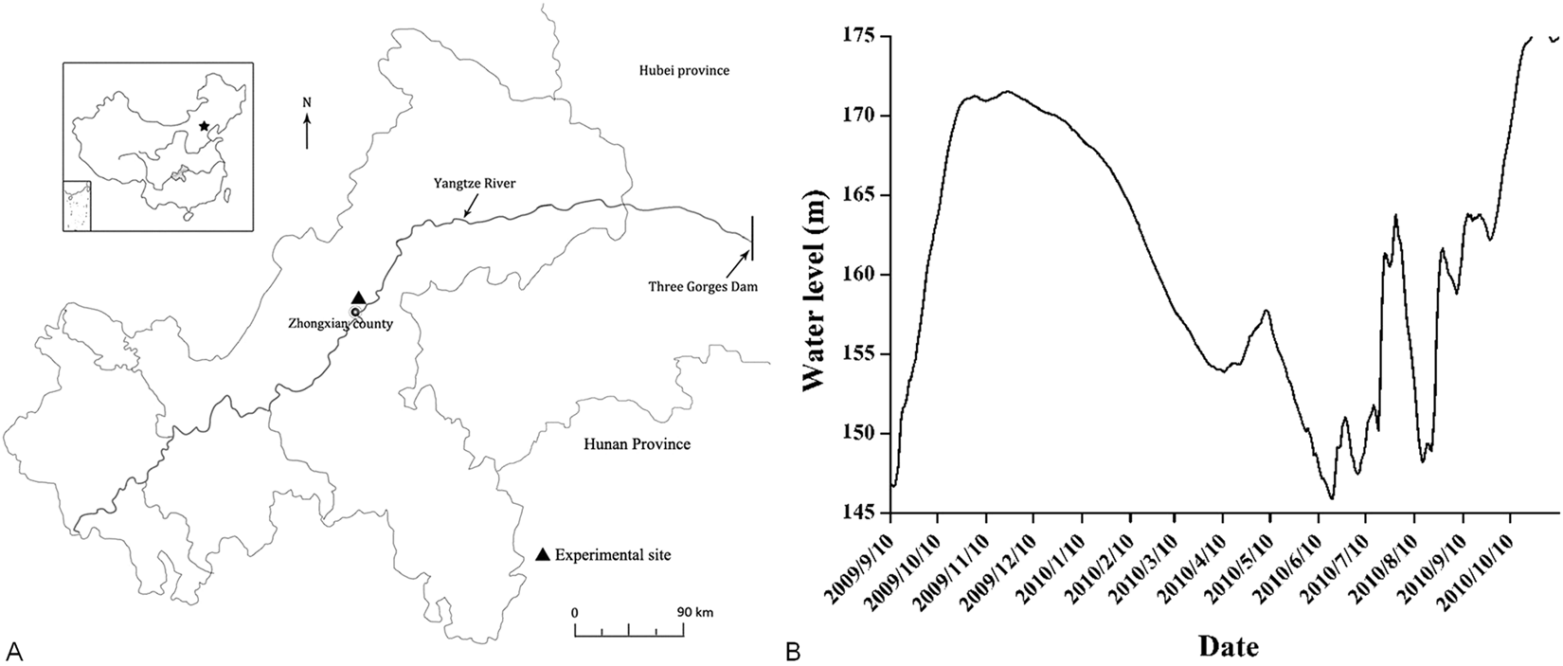
Location and water level variation at the Zhongxian County section of the Three Gorges Reservoir. A, Location of Zhongxian (drawn using ArcGis 10.2 (Esri China Information Technology Co. Ltd., Beijing, China), http://www.esrichina.com.cn/). B, Water level variation at the experimental site between September 10, 2009, and November 20, 2010.

In the TGR WLFZ, the flooding severity during summer has a direct influence on the smooth completion of the production of *X*. *sibiricum* seeds. In July and August 2010, the *X*. *sibiricum* plants at elevations below 165 m did not yield seeds successfully due to accidental flooding (Fig. 5), and the plants at the vegetative period of growth died due to submergence. Taking the exposure duration at different elevations in the TGR WLFZ as well as the maturity of the seeds collected from different elevations in 2009 and 2010 into account, *X*. *sibiricum* plants with an exposure time of more than 130 days can produce GS fruits, and an exposure time longer than 160 days can result in the production of YH and BH fruits (Tables 1, 2 and 4 and Fig. 4). Although the water level in the WLFZ during the flooding season in 2010 increased to 165 m, the normal level is less than 155 m under the current water level scheduling model. Therefore, *X*. *sibiricum* plants growing at elevations of 155 m and higher have a sufficiently long growth phase to produce a sufficient amount of seeds to ensure the continuation of its population in the region.

**Table 4:** Water impoundment date, water recession date and submergence duration at different elevations in the water level fluctuation zone of the Zhongxian County section of the Three Gorges Reservoir from year 2009 to 2010.

Item	Elevation (m)					
	150–155	155–160	160–165	165–170	170–175	>175
Recession date (2009)	2009.05.24–06.01	2009.05.01–05.23	2009.02.09–04.30	2008.12.08–2009.02.08	2008.11.05–12.07	
Impoundment date (2009)	2009.08.04–09.26	2009.09.27–10.03	2009.10.04–10.12	2009.10.13–10.22	2009.10.23–11.24	—
Recession duration (2009) (d)	65–126	128–157	158–246	247–320	321–355	365
Recession date (2010)	2010.03.28–06.04	2010.03.01–03.27	2010.02.07–02.28	2009.12.25–2010.02.06	2009.11.25–12.24	—
Submergence duration (d)	210–260	147–210	117–147	63–117	0–63	0
Impoundment date (2010)	2010.07.11–07.19	2010.07.20–07.22	2010.07.22–08.03	2010.10.04–10.11	2010.10.12–10.27	—

—, this elevation did not experience submergence.

In conclusion, when *X*. *sibiricum* fruits mature to the GH stage, their seeds are already mature and have a high germination ability. *X*. *sibiricum* fruits at different degrees of maturity possess a rather strong ability to withstand submergence and can smoothly complete seed germination. Submergence can enhance seed germination. Under the normal water level scheduling mode, *X*. *sibiricum* plants at elevations above 155 m in the WLFZ have already yielded mature seeds before the impoundment, guaranteeing seed sources for the natural continuation of *X*. *sibiricum* populations in the WLFZ. In the process of plant seed germination, summer flooding, which is an unknown factor, can directly influence whether plants can successfully produce mature seeds. Under the current water level scheduling mode of the TGR, *X*. *sibiricum* can grow and continue its populations naturally in the WFLZ, and therefore, it can be used as a potential species for vegetation restoration and reconstruction in WFLZs.

## Methods and Materials

### Experimental site

The research site is located in the WLFZ in the Zhongxian County section of the TGR (Fig. 5A), Chongqing Municipality, between 107°30′–108°14′E and 30°03′–30°35′N. The habitat at the site is typical of the TGR and has a warm and humid subtropical southeast monsoon climate, with an annual average temperature of 18.2 °C, an annual precipitation of 1200 mm, and a relative humidity of 80%^25^. Figure 5B shows the variation in the water level in the Zhongxian County section of the TGR from September 2009 to November 2010. Table 4 shows the water impoundment in the WLFZ of the Zhongxian County section at different elevations in 2009 and 2010.

### Experimental plants

*X*. *sibiricum* belongs to the genus *Xanthium* of the family Compositae. Its fruit is an achene with a husk formed by involucral bracts. Each fruit has two compartments, and each compartment contains one seed^26^. The two seeds have obvious dimorphic characteristics. The upper seed is smaller, is relatively less mature and has a longer hibernation time. The lower seed is larger, is relatively more mature and has a shorter hibernation time. According to previous field observations, the degree of maturity of *X*. *sibiricum* fruits is assessed according to the color and hardness of the husk. During maturation, the fruit of *X*. *sibiricum* shows the following stages: (1) early maturity, when the husk is green, the texture is soft and the seeds within the fruit have initially taken shape (designated Green Soft (GS)); (2) middle maturity, when the husk is still green but becoming harder and the seeds within the fruit have basically formed (designated Green Hard (GH)); (3) later period of maturity, when the peel is yellow and the texture is hard (designated Yellow Hard (YH)); and (4) full maturity, when the peel is black and hard and the fruit is fully mature (designated Black Hard (BH)).

### Seed germination

#### Seed collection

Seeds were collected from the WLFZ in the Zhongxian County section of the TGR, Chongqing Municipality. When the reservoir started to impound water in September 2009, some *X*. *sibiricum* plants had not yet produced fully mature fruits. The increasing water level during the impoundment caused changes in the degree of maturity of the seeds produced by the plants at different elevations. The degree of maturity of the fruits collected at different elevations also differed. For this reason, during seed collection, we classified the WLFZ into seven elevation gradients with vertical height increments of 5 m: 145–150 m, 150–155 m, 155–160 m, 160–165 m, 165–170 m, 170–175 m, and >175 m. To ensure that *X*. *sibiricum* plants at different elevations had the maximum possible time for growth and reproduction, the seed collection time was set to occur when the water was about to submerge the target elevation. As a result, the fruits with the highest possible degree of maturity at this elevation were collected. The types of *X*. *sibiricum* fruits collected at different elevations are shown in Table 1. Because the *X*. *sibiricum* plants growing at an elevation of 145–150 m were still in the vegetative stage of growth during the impoundment, their fruits were not collected. Fruits at different elevations were collected from individual plants belonging to a variety of *Xanthium* populations on the bank of the reservoir in the WLFZ.

#### Seed submergence treatment

The submergence depth and time differ among elevations in the TGR WLFZ. To study the tolerance of *X*. *sibiricum* seeds to submergence at different elevations, the experiment classified the elevations from 145–175 m in the WLFZ into seven gradients using a vertical height increment of 5 m. Seeds were placed at these elevations, namely 145–150 m, 150–155 m, 155–160 m, 160–165 m, 165–170 m, 170–175 m, and >175 m, and the seeds at an elevation higher than 175 m did not undergo submergence and were thus used as a control. The fruits had to be collected only when the target elevation was to be submerged (for time of seed collection, see Table 1). Therefore, during fruit placement, fruits collected at each elevation were only placed at the collection elevation or higher. The placement pattern is shown in Table 5. The placement time was fixed in accordance with the rising water, specifically when the water was about to inundate the target elevation. One sample consisted of 100 hundred grains of *X*. *sibiricum* seeds (200 seeds), which were placed on the surface soil (in which there were no *X*. *sibiricum* seeds prior to placement) in a plastic pot, and the pot was then covered with a nylon net to ensure that the submerged seeds were not lost and that no foreign seeds invaded the pot. Each treatment consisted of 20 pots.

**Table 5:** Placement of fruits of different maturity states﻿﻿﻿ at different elevations in water-level-fluctuation-zone of the Three Gorges reservoir.

Elevation (m)	Fruit types placed					
145–150	—	—	—	—	—	—
150–155	GS	—	—	—	—	—
155–160	GS	GH	—	—	—	—
160–165	GS	GH	YH160–165	—	—	—
165–170	GS	GH	YH160–165	YH165–170	—	—
170–175	GS	GH	YH160–165	YH165–170	BH170–175	—
170–175	GS	GH	YH160–165	YH165–170	BH170–175	BH

— no fruit was placed. GS indicates green soft fruits; YH160–165 indicates yellow hard fruits collected at 160–165 m; YH165–170 indicates yellow hard fruits collected at 165–170 m; and BH170–175 indicates black hard fruits collected at 170–175 m.

#### Seed germination calculation

After the water recession, the nylon net over the pot was removed. The fruits were watered regularly to maintain the moisture of the soil in the pot. The germination of seeds at different elevations was observed every 10 days until the subsequent submergence at the end of the experiment in 2010.

The accumulated seed germination percentage (G_i_) was calculated according to the formula G_i_ = g_i_/R × 100%, where G_i_ is the germination percentage until the *i*th day, g_i_ is the total germination number by the ith day, and R is the seed number used for the germination experiment. The seed germination percentage (G) is the total germination percentage until the end of the germination period. The germination energy was calculated as the total number of germinated seeds after 45 days divided by the total number of all tested seeds times 100%. This parameter was used to calculate the rate and regularity of seed germination at the early stage, which reflects the vitality of the seeds.

### Seed production

Twenty seedlings were randomly selected from the earliest batch of seedlings germinated from *X*. *sibiricum* seeds from fruits of different maturity state﻿s﻿ placed at each elevation. When the seedlings were 10 cm high, they were transplanted to the corresponding elevation for growth observation. The basic environment (slope direction, east by south 21°; slope gradient, 5.8°; soil type, yellow earth; and soil water-holding capacity, 80–90%) at each elevation was the same. The plants were recovered when the reservoir impoundment was about to submerge the transplanted *X*. *sibiricum* plants at different elevations. The seeds at different degrees of maturity produced by each strain were counted.

### Statistical analysis

The effects of submergence on the seed germination of fruits at different degrees of maturity and on seed production were analyzed through one-way ANOVA. The least significant differences method was used to perform comparisons at the 95% confidence level. The data were transformed to prevent variance heterogeneity. If the transformed data did not satisfy the requirement of homogeneity of variance, a nonparametric test was performed to compare and analyze the data. SPSS 18.0 was used for the statistical analyses of the data, and Origin 8.5 was used to plot the data.
