# Publisher Correction: Effects of water level regulation on the seed germination and production of annual plant *Xanthium sibiricum* in the water-level-fluctuating-zone of Three Gorges Reservoir

**DOI:** 10.1038/s41598-018-29004-6

**Published:** 2018-07-11

**Authors:** Jianhui Liu, Feng Lin, Shaohua Shi, Qiaoli Ayi, Songping Liu, Bo Zeng

**Affiliations:** Key Laboratory of Eco-environments in Three Gorges Reservoir Region (Ministry of Education), Chongqing Key Laboratory of Plant Ecology and Resources Research in Three Gorges Reservoir Region, School of Life Sciences, Southwest University, Chongqing, 400715 China

Correction to: *Scientific Reports* 10.1038/s41598-017-04599-4, published online 11 July 2017

Shaohua Shi and Songping Liu were omitted from the author list in the original version of this Article. This has been corrected in the PDF and HTML versions of the Article.

In addition, in the original version of this Article, Jianhui Liu and Feng Lin were omitted as equally contributing authors.

The Author Contributions section now reads:

“B.Z. conceived and designed the experiment. J.L., F.L., Q.A., S.S., and S.L. performed the experiments. J.L. and B.Z. conducted the data analysis, J.L., Q.A., F.L. wrote the paper. J.L. and F.L. contributed equally to this work.”

